# cAMP-Mediated Modulation of Functions of Green- and Blue-Sensitive Cones in Zebrafish

**DOI:** 10.3390/ijms26167882

**Published:** 2025-08-15

**Authors:** Darya A. Nikolaeva, Luba A. Astakhova

**Affiliations:** Sechenov Institute of Evolutionary Physiology and Biochemistry, RAS, 194223 Saint-Petersburg, Russia

**Keywords:** photoreceptors, cones, cAMP, adaptation, photoreceptors, zebrafish, signal transduction

## Abstract

Although cyclic adenosine monophosphate (cAMP) is not a major secondary messenger in the visual transduction cascade in vertebrates, it may modulate photoreceptor functions. The effects of cAMP have been extensively studied in rods; however, its role in cones remains less understood. The aim of this study was to investigate the effects of increased levels of cAMP on the photoresponses of isolated blue- and green-sensitive cones in adult zebrafish (*Danio rerio*). To examine the effects of elevated cAMP on individual cone spectral types, photoreceptor currents were recorded using a suction pipette method. The adenylate cyclase activator forskolin was used to increase intracellular cAMP levels. Sensitivity and photoresponse parameters were compared before and after forskolin application. An increase in cAMP levels has similar effects on photoresponses of blue- and green-sensitive cones. Forskolin application to both types of cones resulted in a slight increase in sensitivity, with significant slowing of the phototransduction cascade shutdown processes and a marked increase in the integration time of photoresponses. These findings suggest that intracellular cAMP levels, which fluctuate in the retina during the diurnal cycle, can modulate cone function. The observed effects of cAMP are consistent with its action on one of its main putative targets, opsin kinases.

## 1. Introduction

Vision is one of the critical sensory modalities for living organisms, enabling them to respond with high accuracy to changes and events in their environment. At the same time, illumination levels and visual stimuli in a natural context can vary by up to 10 orders of magnitude. The vertebrate visual system can operate across this entire spectrum, at least thanks in part to two types of photoreceptors found in the retina and their associated adaptation mechanisms. There are two main types of photoreceptors in vertebrates: rods and cones. Rods are receptors for night vision, characterized by high sensitivity and slow photoresponse development. Cones are day vision receptors that exhibit rapid photoresponses and lower light sensitivity and do not saturate even at the highest intensities [1,2].

The biochemical cascade of visual transduction is principally shared by rods and cones and involves several amplification steps [3,4]. The visual pigment, a member of the G-protein-coupled receptor family, interacts with a GTP-binding protein called transducin upon absorbing light, producing its active form, T* (Tα-GTP). Each T* molecule activates the effector enzyme cGMP-phosphodiesterase (PDE), significantly increasing the hydrolysis rate of cyclic guanosine monophosphate (cGMP), the secondary messenger in the vertebrate phototransduction cascade. cGMP-gated cation channels located in the outer segment plasma membrane are highly sensitive to a decline in cGMP levels, which causes the cyclic nucleotide-gated (CNG) channels to close. Photoreceptor cells hyperpolarize, and neurotransmitter release from the synaptic terminal slows down. This is the process of the response to light that occurs in the retina. The light-activated cascade is quenched by mechanisms operating at each activation step. Light-induced visual pigment activity is decreased by multiple phosphorylation carried out by opsin kinases (also called G-protein-coupled receptor kinases, GRKs) and subsequent arrestin binding. The active TαGTP-PDE* is turned off by its intrinsic GTPase activity, while the restoration of dark cGMP levels is due to continuously running guanylate cyclase (GC).

The visual system can operate over a wide range of illuminances thanks to light adaptation mechanisms, which occur at various stages of visual perception and processing, primarily at the level of photoreceptor cells [5]. The first and most extensively studied group of these mechanisms relies on intracellular calcium concentrations [6,7]. The regulatory effect of calcium is based on changing the balance of its input and output through the plasma membrane of the photoreceptor outer segment. Under illumination, the concentration of calcium in the cytoplasm decreases. This leads to increased GRK activity via recoverin, increased GC activity via guanylate cyclase activating protein (GCAP), and increased affinity of CNG channels for cGMP via calmodulin. Calcium feedbacks contribute greatly to light adaptation, but it cannot fully explain the range of adaptation in rods and cones. This suggests the existence of additional calcium-independent regulatory mechanisms in the phototransduction cascade, and the search for such additional mechanisms is ongoing.

The search for new regulatory loops in the phototransduction cascade, which underlies light and dark adaptation as well as the adjustment of photoreceptor functions in accordance with diurnal rhythms, led to the discovery of the important role of cyclic adenosine monophosphate (cAMP) in regulating visual transduction. The level of cAMP in vertebrate retinal cells is light-dependent and consequently follows a diurnal rhythm [8,9,10]. Its concentration decreases cyclically during the day and increases at night. This pattern indicates that the intracellular level of cAMP may regulate circadian adjustments in the retina, facilitating adaptation to high illumination levels during the day and low illumination levels at night. Recent work suggests that such cAMP-based regulation may also occur at the level of photoreceptor cells. A number of studies have shown that direct effectors of intracellular cAMP—protein kinase A (PKA) and exchange protein activated by cAMP (EPAC)—have targets among the proteins participating in the phototransduction cascade. These targets include GRKs, whose phosphorylation has been demonstrated in both in vitro and in vivo studies [11,12,13]. Furthermore, it has been shown that cAMP levels influence the function of CNG channels [14], as well as phosducin, a protein whose function is not fully understood but which can bind to the α-subunit of transducin [15,16,17,18]. With regard to the capacity of cAMP to modulate the functions of photoreceptor cells in general, a number of electrophysiological studies have been conducted in recent years. Our group [19] carried out research on amphibian rods and demonstrated that an increase in intracellular cAMP levels, comparable to the daily fluctuations in the retina, causes more than a twofold increase in rod sensitivity to light stimuli. It was also found that pharmacological elevation of cAMP significantly increases the signal-to-noise ratio in amphibian rods [20]. Furthermore, direct measurements of cAMP levels in the outer segments of rods from the same species revealed that saturating background illumination results in an immediate increase in cAMP levels and the simultaneous activation of PKA [21]. However, in mouse rods, another method showed a short-term decrease in PKA activity under saturating illumination, followed by a subsequent increase in PKA activity after the light was turned off [22].

In this context, the question of whether the phototransduction cascade in cones is regulated by cAMP seems non-trivial. Cones are daytime photoreceptors that operate at high levels of illumination; at twilight, their contribution to vision is almost negligible. An increase in sensitivity during the ‘dark’ phase would be functionally unnecessary for them. However, cAMP-dependent regulation in cones could exist independently of circadian rhythms, enabling them to quickly and efficiently adjust to changing light levels during the daytime. Nevertheless, considerably less is known about the ability of cAMP to regulate the phototransduction cascade in cones, and the available data are somewhat contradictory. Previously, our group pharmacologically increased cAMP levels in isolated green- and red-sensitive cones of the golden carp (*Carassius carassius*) and demonstrated that this slows the kinetics of photoresponse shutdown without altering the cones’ sensitivity to light [23]. Another study on the whole eye of five-day-old zebrafish larvae showed that increasing cAMP levels slowed dark adaptation in cones (without differentiation by spectral type), but this was not accompanied by changes in sensitivity to light flashes or photoresponse parameters [24]. The discrepancy between the results obtained for the cones of adult *Carassius carassius* and *Danio rerio* larvae may be due to underdeveloped signaling systems in larval cones or to the effects of mixing of responses from all spectral cell types.

Thus, there is a need to clarify how the regulatory effects of cAMP influence the function, light sensitivity, and photoresponses of mature vertebrate cones of different spectral types in the fully developed retina. Zebrafish (*Danio rerio*) is a suitable species for such a study, as the expression of GRKs in their photoreceptors has been characterized: the cones of this species possess a retinal GRK expression pattern similar to that of humans, and both Grk1b and Grk7 are expressed and participate in returning cone photoreceptors to the dark-adapted state [25].

## 2. Results

In darkness, photoreceptors generate a photocurrent that flows through the cytoplasm, across the membranes of both the outer and inner segments, and through the extracellular space. Light partly or completely stops the photocurrent, depending on the spectrum, intensity, and duration of the stimulation. The suction pipette allows recording of the photocurrent from an individual photoreceptor.

In the present study, we used the suction pipette method to selectively assess the effect of forskolin-induced elevated cAMP levels on isolated fish cones of two spectral classes: green-sensitive (or medium-wavelength-sensitive, MWS) and blue-sensitive (short-wavelength-sensitive, SWS2) cones of zebrafish. We should note that until recently, and as far as we know, no studies had selectively recorded photoresponses from isolated zebrafish cones. The very first research of its kind was published earlier this year [26]. Our group adapted the suction pipette method [27] for zebrafish cones and used it to analyze the effects of forskolin, which is the focus of the present study.

To increase the intracellular level of cAMP, we employed the adenylate cyclase activator forskolin. In our previous study on amphibian rods, forskolin increased cAMP concentration in the outer segment approximately twofold at 2 µM and sixfold at 10 µM. This effect plateaued within approximately 15 min [19]. In the present study, the perfusion solution in the whole sample holder was replaced with a solution containing forskolin; replacement was completed within approximately 3.5 to 4 min. Considering these circumstances, photoresponses were recorded under the ‘forskolin-exposed’ conditions approximately 20 min after the perfusion solution replacement began.

In our previous research on amphibian rods examining the impact of cAMP on photoresponses, recordings were performed using two configurations: ‘outer segment out’, in which outer segments were exposed to drug and inner segments were held in the suction pipette, and ‘inner segment out’, where drug was applied to inner segments while outer segments were in the pipette. Perfusing either the rod’s outer or inner segment with a solution containing forskolin produced comparable effects in both configurations [19,20]. In this work, we used the ‘inner segment out’ configuration to record cone currents. Based on our experience, this approach significantly increased the number of cells that remained viable throughout the entire experimental protocol.

Four morphologically and spectrally different types of cone photoreceptor have been found in the retina of zebrafish: UV-sensitive (SWS1), blue-sensitive (SWS2), green-sensitive (MWS), and red-sensitive (LWS) [28]. In the retina of adult fish, these cone types are arranged into a precise mosaic pattern, characterized by alternating rows of double cones composed of green- and red-sensitive cones, alongside single large blue- and small UV-sensitive cones. Consequently, the retina of this species contains twice as many green- and red-sensitive cones as blue- and UV-sensitive cones. The structural organization of the photoreceptor layer of the zebrafish retina directly determines the composition and cellular ratios of photoreceptor suspensions used for current recordings. For analysis, we have chosen a representative type from both double-cone (the green-sensitive) and single-cone (blue-sensitive) subpopulations.

**Figure 1:**
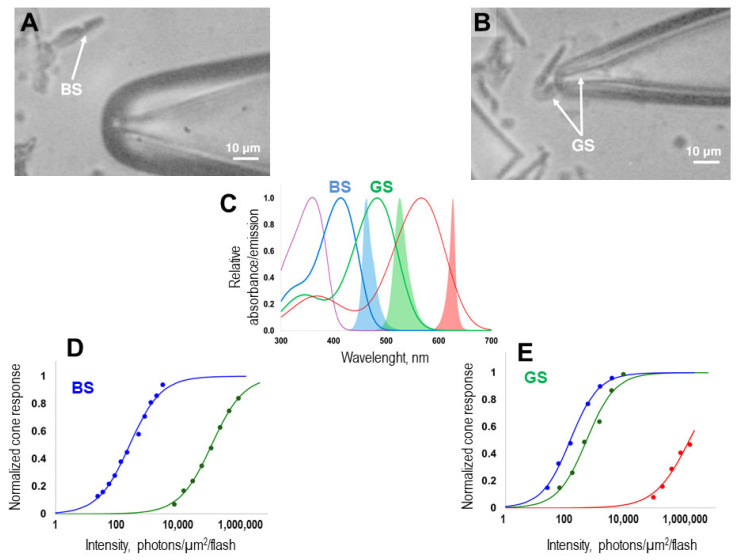
A method for identifying the spectral type of zebrafish cones in a suspension of photoreceptors prepared for the suction pipette technique. The typical morphology of isolated cones is shown below: (**A**) A microphotograph of a blue-sensitive (BS) cone (indicated by a white arrow), representing a single cone with a large inner segment. (**B**) A microphotograph of a green-sensitive (GS) cone (indicated by white arrows), which is one member of a double cone; the outer segment is positioned inside the pipette. Scale bar: 10 μm. (**C**) Overlapping the absorption spectra of zebrafish cone pigments (thin colored curves), calculated by A. Rotov using Govardovskii’s template, based on the absorption maxima from the same study [29] and the emission spectra of LEDs available in our setup (shaded curves in the corresponding colours). (**D**,**E**) Determination of the spectral type of the cone by differential sensitivity to blue, green, and red light flashes (λ_max_ = 460, 525, and 630 nm, respectively). On the panel (**D**) blue symbols and line are response-intensity curve of BS cone to blue light flashes; green symbols and line are response-intensity curve of the same BS cone to green light flashes. On the panel (**E**) blue symbols and line, green symbols and line and red symbols and line are response-intensity curves of one example GS cone to blue, green and red light flashes, respectively.

In the prepared photoreceptor suspension, double cones are more commonly found, whereas single cones sensitive to blue and UV light are less frequently observed. In our experiments, to find a green-sensitive cone, we captured the outer segment of one of the double cones with a pipette. When searching for a blue-sensitive cone, we captured the outer segment of a single large cell. Figure 1A,B show the typical morphology of the cells whose currents we recorded in our study.

In order to accurately identify the spectral type of the cone that was drawn into the pipette, we determined its sensitivity to flashes of light with different spectral compositions. In our setup, three stimulating LEDs were used, each with a specific absorption maximum: red (λ_max_ = 630 nm), green (λ_max_ = 525 nm), and blue (λmax = 460 nm) (for more details, see the Material and Methods section). By overlaying the absorption spectra of the individual cone types of zebrafish with the emission spectra of the LEDs, we were able to precisely estimate the differential sensitivities of green- and blue-sensitive cones (see Figure 1C). The response–intensity curves recorded for each cone at the beginning of the protocol allowed us to accurately assign the cone to its specific spectral type (Figure 1D,E).

After determining the spectral type, the blue- and green-sensitive cones underwent a light stimulation and pharmacological exposure protocol designed to evaluate the potential effects of forskolin, which elevates cAMP levels. For light stimulation of both green- and blue-sensitive cones, we used blue light flashes (λ_max_ = 460 nm). The light stimulation protocol included recording photoresponses to the flashes as follows: saturating flashes (to assess dark current), approximately half-saturating flashes (to analyze sensitivity and the kinetic parameters of the responses in detail), two consecutive saturating flashes at different intervals, and/or a set of saturating flashes with increasing intensity (to determine the rate of the limiting step of the cascade based on the time constant τ_D_ from Peppeberg’s analysis). For each cone, photoresponses were first recorded in Ringer’s solution, and a second set of measurements was conducted 20 min after replacing the normal Ringer’s solution with Ringer’s solution containing 10 µM forskolin.

Control experiments were performed on both types of cones studied: green- and blue-sensitive. In the control group, the isolated cones were subjected to the same experimental protocol as those in the forskolin group. The control cones were incubated in Ringer’s solution containing 0.1% DMSO (solvent) for approximately 20 min. The effects of forskolin on each photoresponse parameter were then assessed by comparison with the control group.

**Figure 2:**
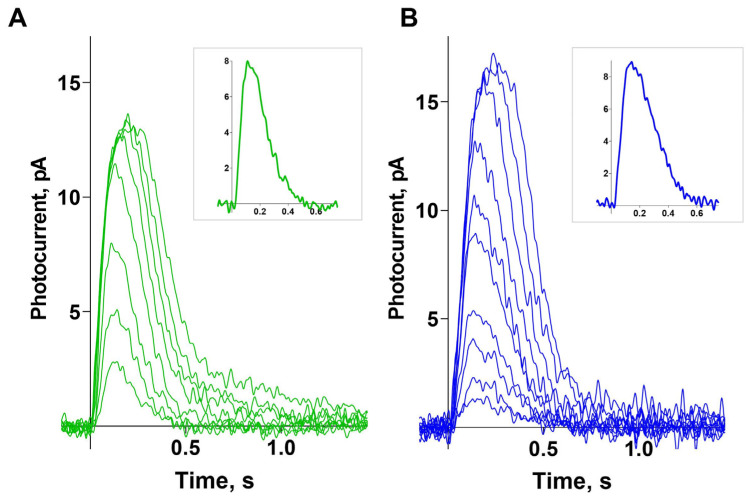
Typical sets of photoresponses from green-sensitive (**A**) and blue-sensitive (**B**) cones to light flashes of increasing intensity in normal Ringer’s solution. The intensities of the flashes for the responses shown in panel A were 24, 56, 1.42 × 10^2^, 5.38 × 10^2^, 1.35 × 10^3^, 3.39 × 10^3^, 6.79 × 10^3^, and 1.36 × 10^4^ photons/μm^2^ per flash, λ_max_ = 460 nm. The intensities of the flashes for the responses in panel B were 24, 36, 89, 1.42 × 10^2^, 5.38 × 10^2^, 8.53 × 10^2^, 1.35 × 10^3^, 3.39 × 10^3^, 6.79 × 10^3^, and 1.36 × 10^4^ photons/μm^2^ per flash, λ_max_ = 460 nm. The insets on panels A and B show typical near-half-saturated responses for GS and BS cones from the presented sets.

Figure 2 (panels A and B) shows typical sets of photoresponses from green- and blue-sensitive cones to blue light flashes of increasing intensity. For the analyzed cells, the mean dark current was 12.7 ± 0.7 pA for green-sensitive (GS) cones and 12.1 ± 0.7 pA for blue-sensitive (BS) cones. Near half-saturated responses differ in shape between GS and BS cones, with BS cones demonstrating slower recovery (the time constants of the recovery phase are 140 ± 11 ms and 184 ± 16 ms, respectively; a significant difference was found using an unpaired *t*-test; both parameters are presented as mean ± SEM). Typical near half-saturated responses for GS and BS cones are shown in the two corresponding insets.

### 2.1. Forskolin Increases the Sensitivity of the Green- and Blue-Sensitive Cones

To examine how forskolin affects cone sensitivity to light, we recorded photoresponses to approximately half-saturating flashes first in normal Ringer’s solution and then again after 20 min of exposure to 10 μM forskolin (or 0.1% DMSO for control experiments). The intensity of the half-saturating flash was selected individually for each cone and varied within a small range due to variations in the sensitivity of the particular cells. For green-sensitive cones, this range was 224–701 photons/μm^2^ per flash, and for blue-sensitive cones, it was 340–548 photons/μm^2^ per flash. These non-saturated responses were then normalized to the dark current, determined by the amplitude of the saturated response recorded in the corresponding closest time point, to obtain the fractional response. As a criterion for possible changes in sensitivity induced by forskolin or in the control group, we used the ratio of the fractional response amplitude after exposure to that before exposure.

**Figure 3:**
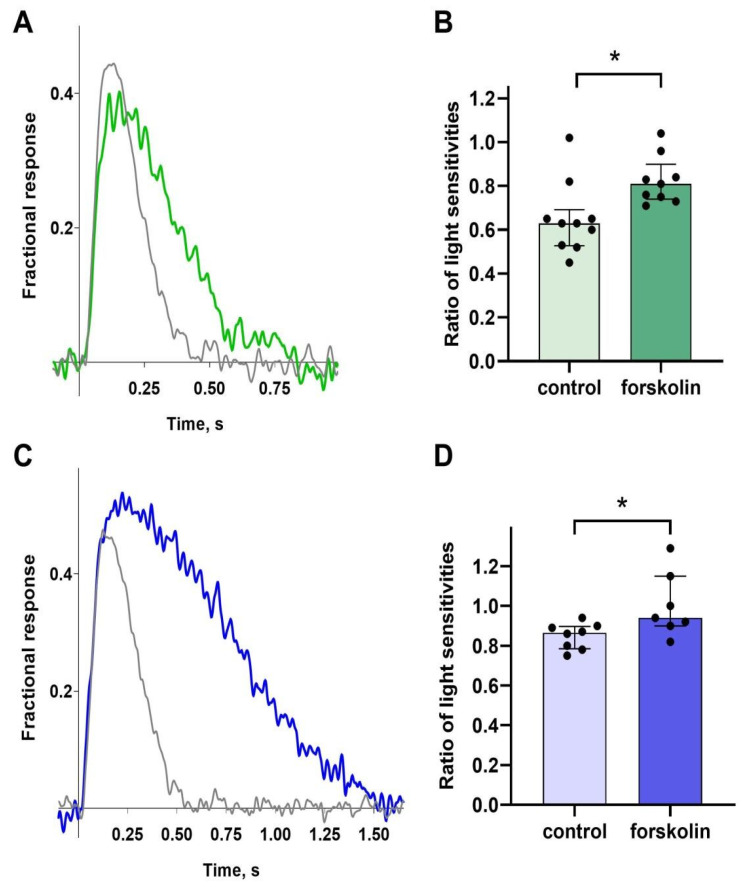
Light sensitivity of green- and blue-sensitive cones before and after forskolin exposure. Fractional, non-saturated responses recorded in normal Ringer’s solution (grey curves) and following 20-min forskolin exposure (green/blue curves) for a GS cone (**A**) and a BS cone (**C**). The intensities of near half-saturated flashes for the responses shown in (**A**,**C**) were 89 and 539 photons/μm^2^ per flash, respectively, λ_max_ = 460 nm for both panels. Comparison of sensitivity ratios between the control and forskolin groups for GS cones (**B**) and BS cones (**D**); data are shown as median ± interquartile range. An asterisk (*) denotes statistically significant differences between groups (Mann–Whitney test; *p*  = 0.007, Cohen’s d = 1.5 for GS cones; *p*  = 0.014, Cohen’s d = 1.6 for BS cones). Typical examples of fractional, non-saturated responses in normal Ringer’s solution and following 20 min exposure to 0.1% DMSO (control groups) for green- and blue-sensitive cones are shown in Supplementary Materials Figure S1.

Exposure to 10 μM forskolin in green-sensitive cones led to a slight decrease in sensitivity (a representative example of normalized responses before and after forskolin exposure is shown in Figure 3A), while the control group exposed to 0.1% DMSO exhibited a greater drop in sensitivity. The difference was statistically significant (see Figure 3B). So, we interpreted this to mean that forskolin increases the sensitivity. A very similar effect of forskolin on sensitivity was observed in blue-sensitive cones (Figure 3C,D), and we also concluded that forskolin increased the sensitivity of blue-sensitive cones compared to controls.

### 2.2. Forskolin Slows Down the Photoresponse Turn-Off Without Affecting the Activation Phase in Green- and Blue-Sensitive Cones

Visual comparison of responses before and after forskolin treatment reveals a noticeable change in the kinetics of photoresponses, which, at first glance, seems to be related to the inactivation phase of the phototransduction cascade. For a more rigorous quantitative assessment of the kinetic parameters of photoresponses affected by forskolin, we performed a mathematical analysis of potential changes.

**Figure 4:**
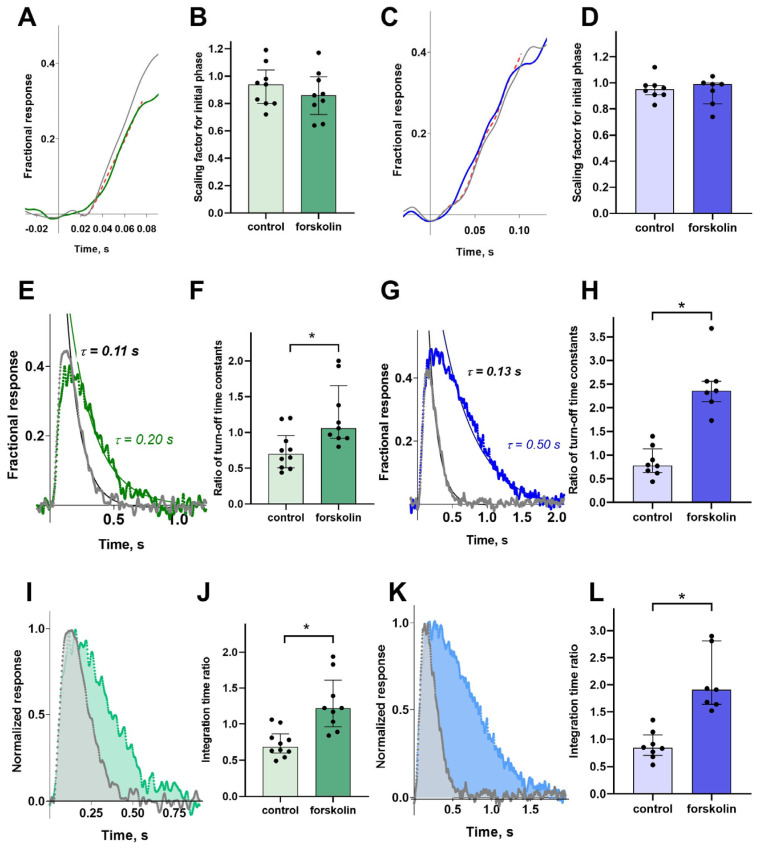
The effects of forskolin on different parameters of non-saturated responses of green- and blue-sensitive cones. Estimation of potential change of the initial phase of the response using a scaling factor for non-saturated responses of a green- (**A**) and a blue-sensitive (**C**) cones (flash intensities 89 photons/μm^2^ per flash for a green-sensitive cone and 427 photons/μm^2^ per flash for a blue-sensitive cone, λ_max_ = 460 nm for both cases). In (**A**,**C**), the grey solid line shows the response in normal Ringer’s solution, and the green/blue solid line depicts the response after forskolin exposure in green-sensitive and blue-sensitive cones, respectively. The red dotted line shows the response in normal Ringer’s solution multiplied by a scaling factor to best approximate the response after forskolin exposure. (**B**,**D**) present a comparison of the scaling factors for the initial phase between the control and forskolin groups for green-sensitive and blue-sensitive cones, respectively. Fitting the falling phase of the unsaturated response with an exponential function for green- (**E**) and blue-sensitive (**G**) cones before and after forskolin exposure. Comparison of the ratio of the recovery time constants after/before forskolin exposure between the control and forskolin groups for green- (**F**) and blue-sensitive (**H**) cones. Impact of forskolin exposure on the integration time of non-saturated responses for a green-sensitive (**I**) and a blue-sensitive (**K**) cones. Comparison of the integration time change between the control and forskolin groups for green-sensitive (**J**) and blue-sensitive (**L**) cones. Data are shown as median ± interquartile range. The presence of an asterisk (*) signifies statistically significant group differences determined by the (Mann–Whitney test; *p*  = 0.008, Cohen’s d = 1.5 for turn-off rate of GS cones; *p*  = 0.0003, Cohen’s d = 3.1 for for turn-off rate of BS cones; *p*  = 0.0004, Cohen’s d = 2.3 for integration time of GS cones; *p*  = 0.0003, Cohen’s d = 3.1 for integration time of BS cones).

To estimate the possible effect of forskolin on the activation rate of the phototransduction, we evaluated the variability of the initial phase of the response. Cones are characterized by extremely rapid processes in the phototransduction cascade, including very fast turn-off processes, which begin to affect the response curve shape from the very first fractions of a second. Therefore, we suppose that fitting the initial phase with a simple mathematical function, for example, a quadratic polynomial, as done in some studies [30,31], can introduce significant errors in calculations. For the purposes of our study, we only needed to determine the relative changes in the steepness of the initial phase. Therefore, we extracted the ratio of the two activation rates by adjusting the rising phases of two normalized photoresponses, recorded before and after exposure with a scaling factor (Figure 4A,C).

Visual inspection of the two normalized unsaturated responses, pre- and post-forskolin treatment, indicates a slowing of the photoresponse turn-off. To quantify this effect and assess its significance, we fitted the falling phase of the photoresponse with an exponential function, as shown in Figure 4E,F for the green- and blue-sensitive cone responses, respectively. This fit provides a photoresponse turn-off rate constant. It is observed that the time constant increased with forskolin treatment in both the green- and blue-sensitive cones; that is, the rate of photoresponse turn-off slowed. This effect is significant compared to the control group, as shown in Figure 4F,G, especially since the control group and/or the duration of the experiment conversely accelerate the photoresponse turn-off time constant.

We also analyzed how forskolin affected the integral parameter—the integration time of the non-saturated response, which depends on the main kinetic parameters of the response curve (see Figure 4I,K). The analysis showed that, compared to the control, forskolin leads to a significant and pronounced increase in the integration time in both green- and blue-sensitive cones.

### 2.3. Forskolin Slows Down the Dominant Shut-Off Time Constant

**Figure 5:**
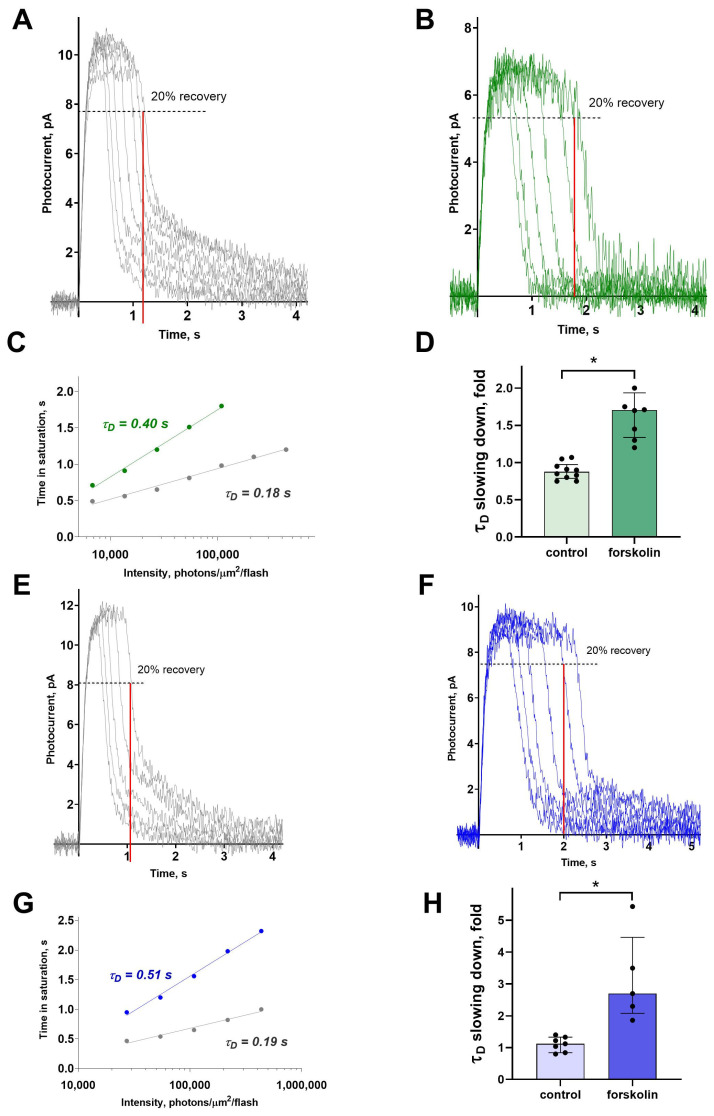
The effects of 10 μM forskolin on dominant shut-off time constant in green- and blue-sensitive cones. The sets of photoresponses from a green-sensitive cone to saturating flashes of increasing intensity in Ringer’s solution and after exposure to forskolin, in (**A**) and (**B**), respectively. Time in saturation (vertical red solid line) is determined in the point of 20% current recovery (black dashed horizontal line). (**C**) Pepperberg’s analysis and the determination of τ_D_ for the same green-sensitive cone. Grey circles show the dependence of saturation time on flash intensity in Ringer’s solution, green circles—the same dependence after forskolin exposure. The grey and green solid lines represent logarithmic function approximation that determines the characteristic time constants, τ_D_. The sets of photoresponses from a blue-sensitive cone to saturating flashes of increasing intensity in Ringer’s solution and after exposure to forskolin in (**E**) and (**F**), respectively. (**G**) Pepperberg’s analysis and the determination of τ_D_ for the same blue-sensitive cone. Grey circles show the dependence of saturation time on flash intensity in Ringer’s solution; blue circles—the same dependence after forskolin exposure. The grey and blue solid lines represent logarithmic function approximation that determines the characteristic time constants, τ_D_. Estimation of the impact of forskolin exposure on the recovery constant, τ_D_, for green- (**D**) and blue-sensitive (**H**) cones versus the control groups. Data are shown as median ± interquartile range. Asterisk (*) marks statistically significant difference between groups based on the Mann–Whitney test; *p*  = 0.00005, Cohen’s d = 3.1 for GS cones; *p*  = 0.003, Cohen’s d = 2.9 for BS cones.

To estimate the impact of forskolin-induced cAMP elevation on the limiting recovery process, we recorded a series of photoresponses to saturating light stimuli of increasing intensities and analyzed the slope of the Pepperberg line, which represents the relation between time in saturation and flash intensity. This kind of analysis allows evaluation of the rate of the slowest inactivation process in the phototransduction cascade, characterized by the so-called dominant shut-off time constant, or τ_D_ [32]. For plotting such dependence, the time in saturation is defined at the point where 20% of the dark current has recovered, as illustrated in Figure 5A–D. The slope of the curve is determined on a logarithmic scale or from equation T_rec_ = C + τ_D_ ln [I], where I is intensity of the light stimulus and τ_D_ is the dominant shut-off time constant (See Figure 5C,G). Figure 5 illustrates representative examples of τ_D_ estimation for green- and blue-sensitive cones in normal Ringer’s solution and following forskolin exposure. The results indicate that forskolin markedly prolonged the time in saturation, steepened the slope of the Pepperberg line, and consequently increased τ_D_ in both green- and blue-sensitive cones. For both cone types, this effect was significant compared to the control groups (Figure 5D,H).

### 2.4. Forskolin Has No Effect in a Dual Flash Paradigm in Isolated Green- and Blue-Sensitive Cones

**Figure 6:**
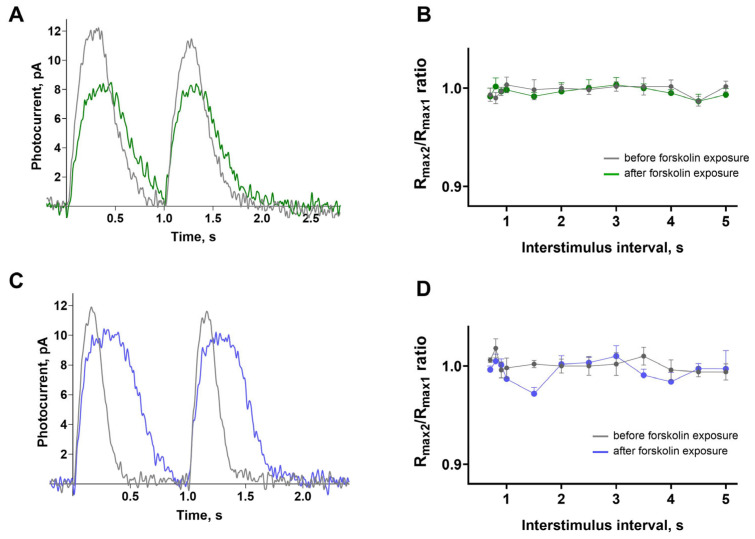
Forskolin has no effect in a dual flash paradigm in isolated green- and blue-sensitive cones. (**A**) A representative response of a green-sensitive cone to two saturating light flashes (3.4 × 10^3^ photons/μm^2^ per flash, λ_max_ = 460 nm, averaged from three curves) with an interstimulus interval (ISI) of 1 s: grey line shows responses in Ringer’s solution, green line shows responses after forskolin exposure. (**B**) Ratios of the response amplitudes of green-sensitive cones to the second stimulus relative to the first are shown across interstimulus intervals ranging from 0.7 to 5 s. Grey symbols represent responses in Ringer’s solution, green symbols represent responses after forskolin exposure. Data are shown as means ± SEM. Sample size *n* = 6. There are no significant differences between before/after forskolin exposure. (**C**) A representative response of a blue-sensitive cone to two saturating light flashes (5.4 × 10^4^ photons/μm^2^ per flash, λ_max_ = 460 nm, averaged from three curves) with an ISI of 1 s: grey line shows responses in Ringer’s solution, blue line—responses after forskolin exposure. Sample size n = 5. (**D**) Ratios of the response amplitudes of blue-sensitive cones to the second stimulus relative to the first are shown across ISI from 0.7 to 5 s. Grey symbols indicate responses in Ringer’s solution, blue symbols indicate responses after forskolin exposure. Data are shown as means ± SEM. Sample size *n* = 5. 5. There are no significant differences between before/after forskolin exposure. Data were analyzed using the Mann–Whitney test, with a significance threshold of *p* < 0.05.

In a previous study by Chrispel et al., the effect of forskolin on the electroretinogram of the larval zebrafish retina was assessed, and the only significant effect found was in a dual flash paradigm [24]. The authors gave two consecutive saturating flashes at intervals, ranging from 0.75 to 5 s, and observed that forskolin impaired the ability of the response amplitude to recover with the second flash. To determine whether this effect also occurred in isolated cones from adult zebrafish, we nearly replicated this protocol exactly: presenting identical saturating stimuli at intervals ranging from 0.7 to 5 s in Ringer’s solution, followed by approximately 20 min incubation in forskolin. Figure 6A,C show examples of responses of the green- and blue-sensitive cones to two successive identical saturating stimuli with a 1 s interval. It is evident that the amplitude of the response to the second flash is practically unchanged. Figure 6B,D illustrate the average ratios of the amplitudes of the responses to the second flash compared to those of the first flash. It is clear that within this range of the interstimulus interval (ISI), the response is completely restored, and forskolin has no effect. Thus, under our experimental conditions, the effect of forskolin reported by Chrispel et al. could not be observed.

### 2.5. The Regulatory Effects of Forskolin Are More Pronounced in Blue-Sensitive than in Green-Sensitive Cones

When we analyzed the effect of forskolin on the kinetic parameters of non-saturated responses and the dominant shut-off time constant from saturated responses, it seemed to us that, for most of the observed effects, the degree of expression was greater in blue-sensitive cones than in green-sensitive cones. In order to test this assumption more rigorously, we performed a direct statistical comparison of the mean changes in the photoresponse turn-off rate, integration time, and dominant shut-off time constant induced by forskolin (i.e., the ratios of each parameter after versus before forskolin treatment) for green- and blue-sensitive cones. We had previously verified that the mean changes in these parameters in the control groups did not show significant differences between green- and blue-sensitive cones. A direct comparison of the mean changes confirmed our assumption: the effects of forskolin on the turn-off rate, integration time of the unsaturated response, and dominant shut-off time constant of saturated responses were statistically significantly more pronounced in blue-sensitive cones. These comparisons between green- and blue-sensitive cones are shown in Supplementary Material Figure S2.

In summary, the main observations from the results described in this section are as follows. Forskolin increases cAMP levels in the outer segments of the cones, making the green- and blue-sensitive cones more sensitive to light. This is accompanied by a slowdown in the rate of photoresponse turn-off and integration time, as well as an increase in the dominant shut-off time constant in responses to saturating stimuli. The activation rate of the phototransduction cascade is unaffected by forskolin. The listed effects in green- and blue-sensitive cones are in the same direction, but the effects themselves are stronger in blue-sensitive cones.

## 3. Discussion

### 3.1. Forskolin as a Tool for Increasing Intracellular cAMP Levels in Isolated Cones

In the present study, we used the adenylate cyclase activator forskolin at a concentration of 10 μM in order to increase the intracellular cAMP levels and to investigate the physiological effects of this increase on the phototransduction cascade in cones of different spectral types. The question of whether incubation of the inner segment of the cone with forskolin at this concentration actually produces the expected effect on cAMP requires special discussion. Overall, we consider this approach to increasing intracellular cAMP levels to be adequate based on the following facts. First, in our previous work [19], we thoroughly demonstrated that incubation of the retinas of the marsh frog with forskolin leads to a 2- and 6-fold increase in the cAMP concentration in the outer segments of rods over a 16 min interval when exposed to forskolin at a concentration of 2 and 10 μM, respectively. In the mentioned study, however, the retinas were incubated with forskolin as a whole, without restricting its access to the outer segments. Yet, in our subsequent work [20], we showed that the physiological effects on photoresponses were equivalent whether or not forskolin was applied to the outer or inner segment, provided that the remaining rod segment was fixed in a suction pipette throughout the experiment. This is a strong argument in favor of the fact that forskolin, even when interacting with the inner segment, can trigger an increase in the concentration of cAMP and subsequent reactions to this increase in the outer segment of the photoreceptor. Another argument supporting the idea that forskolin, when acting on cones, triggers its effects specifically through an increase in cAMP is provided by the results of Chrispell et al. [24], where incubation of whole larval zebrafish in 50 μM forskolin led to phosphorylation of GRK1 in cones. This effect can be attributed precisely to the increase in cAMP levels and its subsequent activation of PKA, rather than to any off-target effects of forskolin.

### 3.2. Targets of cAMP-Mediated Regulation in the Cone Phototransduction Cascade and Their Relationship to the Observed Effects

In the present study, we have shown that in isolated green- and blue-sensitive cones, forskolin (which we believe increases the cAMP level) leads to a slowing down of the turn-off of the non-saturated photoresponse, accompanied by an increase in the integration time, as well as a slowing down of the dominant shut-off time constant. At the same time, the rate of activation of the cascade under the action of forskolin does not change. This can be interpreted as cAMP-mediated regulation (s) primarily affecting the processes involved in the inactivation of the cascade contributors. There is evidence that putative targets of cAMP in the phototransduction cascade include GRKs, whose phosphorylation has been demonstrated in both in vitro and in vivo studies [11,12,13], CNG channels of the outer segment plasma membrane [14], and phosducin [15,16]. There are grounds to believe that the observed effects of slowing down the cascade inactivation processes can be explained completely or largely by the regulatory effect of cAMP on GRKs. In zebrafish cones, unlike rods, two forms of GRK are expressed: GRK1b and GRK7a [33]. Phosphorylation of GRKs occurs via the main effector, protein kinase A (PKA), when the intracellular cAMP levels increase, which naturally happens during the night [11,12,13]. The PKA substrate on the GRKs molecule is the serine residues at the N-terminus, and their phosphorylation leads to attenuation of catalytic activity [11]. Since, in our study, the level of cAMP was also artificially increased, the effects on GRKs via PKA should have had the same direction as during the transition of photoreceptors to the dark phase of the day. Therefore, this should have led to their phosphorylation, a decrease in catalytic activity, and, as a consequence, less effective inactivation of excited visual pigments. This is in excellent agreement with the slowdown in the shutdown of the photoresponse that we observed.

Interestingly, in their specific manifestation, our results differ from the electrophysiological data obtained by Chrispell et al. on zebrafish larvae [24]. In the work of Chrispell et al., non-saturated cone ERG responses recorded from the whole eye of the larva did not change their kinetics under forskolin exposure, and sensitivity to light remained unchanged. At the same time, the authors observed a decrease in the recovery of the cone photoresponse, which was evident in the paradigm of two saturating flashes, i.e., the response to the second flash was several times smaller than the response to the first flash, especially at short ISI. In the present study, we failed to detect the effects of forskolin in the dual flash paradigm. However, we believe that there is no significant contradiction here because both the decreased recovery of the cone photoresponse in the dual flash paradigm reported by Chrispell et al., and the slowing of the turn-off kinetics for single non-saturated photoresponse, along with the increased integration time observed in the present study, may reflect a less efficient and less rapid recovery of the dark state of the visual pigment when the cAMP levels are elevated. Differences in specific electrophysiological manifestations of this phenomenon may be associated either with the age of the animals (we worked on adult fish, while Chrispell et al. studied 5 dpi larval fish) or with the features of the experimental procedure (we identified specific spectral types of cones separately, whereas in Chrispell et al., all spectral types of cones, including UV-sensitive ones, which may be the dominant type in larval fish, could contribute to the photoresponse).

The effect of increasing cAMP on slowing down the dominant shut-off time constant should be discussed separately. In different types of photoreceptors and across various animal species, the rate-limiting process of switching off the cascade may involve different reactions. For example, the phosphorylation of the visual pigment by GRKs does not dominate the time course of rod recovery under normal conditions in species such as mice and humans, whose photoreceptors are rather slender [34]. However, in species with larger photoreceptors, such as salamanders and frogs, the inactivation of the visual pigment, i.e., its phosphorylation or binding with arrestin, is rate-limiting for recovery [35,36]. There are significantly less data on the nature of the dominant shut-off time constant in cones. Thus, in the only work known to us [37], an assessment of the dominant shut-off time constant in isolated salamander cones allowed the authors to conclude that the process of dominating recovery of the bright flash response represents quenching of the active Meta II form of the cone photopigment, and this quenching includes phosphorylation by GRKs. It is difficult to say whether these data can be extrapolated to zebrafish cones. The extent of the slowing τD under the action of forskolin is even greater than the extent of the slowing of the shut-off time constant of the unsaturated response. One suggestion is that in green- and blue-sensitive cones of zebrafish under normal conditions, the dominant shut-off time constant is determined not by phosphorylation of the visual pigment but by another process, and with an increase in cAMP, the phosphorylation process slows down so much that it becomes time-limiting, which could explain the dramatic increase in τD in green- and especially in blue-sensitive cones of zebrafish observed in the present study under the exposure of forskolin.

### 3.3. Regulatory Effects of cAMP Are More Pronounced in Green-Sensitive Cones than in Blue-Sensitive Cones in Zebrafish

In present study, we made a direct comparison of the mean effects of forskolin in green- and blue-sensitive cones. In blue-sensitive cones, the changes in the turn-off rate, integration time of the unsaturated response, and dominant shut-off time constant under forskolin exposure were statistically significantly more pronounced. This suggests that cAMP-dependent regulatory mechanism(s) are more prominent in blue-sensitive cones of zebrafish. What could be the reasons for this?

The zebrafish retina contains distinct blue-sensitive and green-sensitive cones that express specific opsins. The blue-sensitive cones are long single cones expressing only one variation of opsin (SWS2, opn1sw2 gene) with peak sensitivity around 415 nm [38,39]. The green-sensitive cones are members of double cones and express four variants of opsins (RH2-1-RH2-4, opn1mw1-opn1mw4 genes), which arose due to gene duplication events [39,40,41]. These opsins have distinct amino acid residues that influence their spectral tuning, shifting their peak light absorption to different wavelengths between approximately 467 and 505 nm. Specifically, the spectral shifts observed between the RH2-1 and RH2-4 can be attributed to differences in the amino acid residues at positions E113, E122, and E181, which influence the chromophore interaction with opsin, the size and shape of the binding pocket, and the hydration pattern around the chromophore [42]. The multiple green-sensitive opsin genes exhibit distinct spatial expression patterns across the retina of adult zebrafish: the shorter wavelength subtypes (RH2-1 and RH2-2) are expressed in the central to dorsal retina, while the longer wavelength subtypes (RH2-3 and RH2-4) are expressed in more peripheral and ventral areas [41,43]. This spatial differentiation possibly allows zebrafish to optimize their color vision by adapting to different light conditions in their aquatic environment.

Although the blue-sensitive and green-sensitive cones express different opsins, both cone types share the canonical vertebrate phototransduction cascade involving light-induced opsin activation, transducin-mediated phosphodiesterase stimulation, cGMP hydrolysis, and subsequent closure of membrane cyclic nucleotide-gated ion channels [39]. Differences at the molecular and functional levels related to G-protein signaling and phototransduction regulation may underlie the distinct response kinetics and sensitivity observed between blue-sensitive and green-sensitive cones (our results and [26]). Particularly, the two cone arrestins, Arr3a and Arr3b, are found in different types of zebrafish cones—Arr3a is specifically expressed in green- and red-sensitive cones, while Arr3b is exclusively expressed in blue- and UV-sensitive cones—suggesting that they play distinct roles in regulating the kinetics of photoresponse recovery [43,44]. Besides that, the variations in expression and activity of G-protein-coupled receptor kinases (GRKs), specifically GRK1b and GRK7a, between blue-sensitive and green-sensitive cones can influence G-protein signaling indirectly by modulating the rate of opsin deactivation, thereby contributing to differences in the photoresponse properties of these cones [25,33,39].

Together, while the fundamental G-protein signaling pathway in zebrafish blue-sensitive and green-sensitive cones is largely conserved, slight differences in the expression of gene paralogs and regulatory proteins, including arrestins and GRKs, result in distinct functional characteristics between these cone types and could explain why we revealed more pronounced cAMP-mediated regulatory effects in the blue-sensitive cones.

### 3.4. Putative Non-cAMP-Mediated Effects of Forskolin

In the present study, we used forskolin to increase the intracellular cAMP level and to investigate the effects of this increase on the cone phototransduction cascade. One potential limitation of our study could be related to the direct, non-cAMP-mediated effects of forskolin on photoreceptor physiology. For example, it is known that at a concentration of 50 μM, forskolin significantly accelerated desensitization of the GABA A and glycine receptors in amacrine-like cells of the crucian carp retina [45]. It was also shown that in addition to its action on adenylate cyclase, forskolin directly altered the gating of voltage-dependent potassium channels from a clonal pheochromocytoma cell line [46]. A concentration-dependent effect was shown, which became noticeable at concentrations above 50 μM. In our study, we used a forskolin concentration of 10 μM, which is lower than the concentrations that cause the non-cAMP-mediated effects described above. Separate experiments using high concentrations of forskolin were not conducted, since large samples with different concentrations of forskolin would be required to separate the potential direct effects of forskolin on ion channels and the cAMP-mediated effects of forskolin on GRK and other targets, and the study of non-cAMP-mediated effects of forskolin is beyond the scope of this study. We believe that the main contribution to the physiological changes observed in zebrafish cones is due to cAMP-mediated mechanisms.

### 3.5. Translational Considerations in Relation to the Discovery of cAMP-Mediated Regulation in Fish Cones

The cAMP-mediated regulation of cones, as demonstrated by our group in adult fish and by Chrispell et al. in fish larvae [24], probably exists in other species of fish and other vertebrates with developed cone vision. When translating these findings, it is important to consider the expression profile of GRKs in the cones of different species. Similar expression profiles have been observed in carp, humans and other primates, chickens, and spadefoot toads (see [47] for a review), suggesting that these species may have similar cAMP-mediated adaptation mechanisms in their cones. This gives us hope that similar mechanisms operate in human cone photoreceptors and that our knowledge can be applied to studying the pathophysiological mechanisms of diseases associated with impaired GRK expression, such as Oguchi disease [48] and enhanced S-cone syndrome [49].

However, several species have different GRK expression profiles in their cones. For example, only GRK7 is expressed in the cones of dogs and pigs, whereas only GRK1 is expressed in mice. Consequently, the cAMP-mediated regulation of the phototransduction cascade in the cones of these species, implemented through GRKs, may, therefore, have unique features that warrant separate study.

## 4. Materials and Methods

### 4.1. Experimental Animals and Ethics Statement

Adult zebrafish (*Danio rerio*) were obtained from a local supplier and housed in laboratory aquariums at a density of 4 to 6 fish per 15 L. The fish were fed dry fish food and kept under a controlled 12 h light and 12 h dark cycle. For this study, only adult fish, at least 6–8 months old, of both sexes were used. The fish were dark-adapted overnight prior to each experiment to avoid disrupting their usual day–night cycle. They were then euthanized by decapitation, after which the eyes were enucleated, and a piece of the retina was carefully extracted under dim red light using binocular magnification. The remaining retina and the second eyecup were stored in a light-proof chamber at 4 °C prior to use. Throughout the preparations and electrical recordings, the temperature in the experimental room was consistently maintained between 18 and 22 °C.

The handling of experimental animals complied with the requirements of European Directive 2010/63/EU and the recommendations of the Bioethics Committee of Sechenov Institute of Evolutionary Physiology and Biochemistry of the Russian Academy of Sciences (Permit#1-1/2024 of 31 January 2024, issued by the Bioethics Committee of IEPhB RAS).

### 4.2. Solutions and Specimen Preparation

Ringer solution, used for preparation, storage, and perfusion of samples, contained in mM: NaCl 102, KCl 2.6, MgCl2 1, glucose 5, CaCl2 1, NaHCO3 28, HEPES 5, and the pH was adjusted to 7.8–8.0. The forskolin-containing solution was prepared by diluting a fresh 10 mM stock solution in DMSO with Ringer’s solution to achieve a final concentration of 10 µM. All the chemicals were purchased from Sigma-Aldrich (St. Louis, MO, USA). A piece of the isolated retina was placed into a drop of Ringer’s solution, carefully shredded into small fragments using two fine forceps, and gently pipetted several times to isolate individual photoreceptors. Finally, the resulting suspension, consisting of retinal fragments and isolated photoreceptors, was carefully transferred into the perfusion chamber of the experimental setup for subsequent photoreceptor current recordings.

### 4.3. Single-Cell Current Recordings and Determining Cone Spectral Type

To record the photocurrent of isolated photoreceptors, we used Baylor’s original suction pipette technique [27], which we have modified and adapted to our own needs [2,36]. As mentioned above, the zebrafish’s retina contains four morphologically and spectrally distinct types of cones, enabling the fish to detect light wavelengths ranging from UV to red. Our experimental setup allows us to identify three of them (blue-, green-, and red-sensitive cones) by rapidly changing the spectrum of the stimulating light. The light stimulation system of the setup consisted of two independent channels, each based on high-output LEDs. The intensity of both channels was regulated under computer control, adjusted stepwise by neutral density (ND) filters inserted into the beam and continuously by the LED output. The first channel was equipped with an LED emitting at λ_max_ = 525 nm (green), while the second channel featured switchable LEDs with λ_max_ = 460 nm, 525 nm, and 630 nm (blue, green, and red, respectively). The processes of data acquisition, stimulus timing, and flash intensity were precisely controlled using LabView16.0 hardware and software (National Instruments, Austin, TX, USA).

A reliable method for distinguishing spectral types of photoreceptors involves averaging their responses to non-saturating flashes of various colors, followed by a comparative analysis of their sensitivity. Using this approach, we were able not only to identify the spectral types of cones but also to determine the optimal wavelengths for light stimulation. We revealed that both green-sensitive and blue-sensitive cones are more responsive to blue light LED in our setup (λ_max_ = 460 nm) (see Figure 1D,E). Therefore, to stimulate green- and blue-sensitive cones, we used blue light LED. Regarding the duration of the test flashes, we typically used 2 ms flashes to record non-saturated responses. However, when recording saturated responses, particularly supersaturated responses for Pepperberg’s analysis, we gradually increased the flash duration to enhance the overall stimulus luminance (using durations of 4, 8, 16, 32, and 128 ms). For all analyses and in the figure captures, we used the resulting stimulus intensities adjusted for stimulus duration.

### 4.4. Experimental Protocol, Data Processing, and Statistical Analysis

Zebrafish cones were individually sucked into a glass pipette, with their outer segments facing inward, and their responses were recorded under various light conditions. For each cell, responses were initially recorded in Ringer’s solution, and then recorded again 25 min after replacing the Ringer’s solution with 10 µM forskolin. In control experiments, 0.1% DMSO solution was used as a vehicle control in place of forskolin.

A typical light stimulation protocol started by recording responses to non-saturating light stimuli across various wavelengths to identify the spectral type of each cone. Once the spectral type of each cone had been determined, it was stimulated with light flashes (of blue light) that elicited both saturated and fractional responses, the latter being approximately half-saturated. Some cones were exposed to a series of saturating light flashes with gradually increasing intensities, and their responses were recorded to perform the Pepperberg analysis [32]. A dual flash protocol, similar to that described in [24], was applied to other cones to analyze their responses to successive saturating flashes delivered at specified time intervals. All photoresponses of cones were recorded with a 30 Hz low-pass filter and digitized at 2 ms intervals.

Data acquired from the setup were processed using custom software developed in LabVIEW 16.0 and Python 3.8. To assess potential changes in the response characteristics of cones, we examined the following parameters: the dark current, sensitivity, activation rate, turn-off time constant for non-saturating response, and dominant shut-off time constant, τ_D_ (Pepperberg constant).

The sample size (n) represents the number of individual cones analyzed. For each experimental group, including controls, sample sizes ranged from 8 to 10, consistent with standard practice in our research area. From each retina of a single eye, we prepared one or two specimens for suction pipette recordings. As a result, some experiments allowed recording from up to four cells per fish. However, frequently, some cells did not survive the entire experimental protocol and were excluded from the analysis. In total, data for each group were collected from at least three zebrafish, typically four to five. Control and forskolin experiments were performed in an alternating manner, with animals randomly assigned to groups to ensure unbiased distribution.

Statistical analyses were carried out using OriginPro 2021 software. To evaluate the significance of differences between groups, the unpaired t-test or the Mann–Whitney U test was applied. A *p*-value threshold of 0.05 was used to determine statistical significance. The results represent the ratio of the measured parameters following a 20 min incubation of cones in either a forskolin-containing solution or, for the control, a 0.1% DMSO solution, compared to those obtained in normal Ringer’s solution. We used Cohen’s d to determine the effect sizes of the measurements, where a value of d = 0.20 corresponds to a small effect, d = 0.50 to a medium effect, and d = 0.80 to a large effect, following conventional benchmarks in statistical analysis.

## Data Availability

The raw data can be made available by the corresponding author upon written request.

## Supplementary Materials

The following supporting information can be downloaded at: https://www.mdpi.com/article/10.3390/ijms26167882/s1.

## Abbreviations

The following abbreviations are used in this manuscript:

cAMP	cyclic adenosine monophosphate
cGMP	cyclic guanosine monophosphate
GRK	G-protein-coupled receptor kinases
GC	guanylate cyclase
PDE	phosphodiesterase
CNG	cyclic nucleotide-gated channels
PKA	protein kinase A
EPAC	exchange protein activated by cAMP
LED	light-emitting diode
BS	blue-sensitive
GS	green-sensitive
