# Supplementary material for: cAMP-Mediated Modulation of Functions of Green- and Blue-Sensitive Cones in Zebrafish
Source: Int J Mol Sci. 2025 Aug 15;26(16):7882. doi: 10.3390/ijms26167882 (PMC12386505; doi:10.3390/ijms26167882)
Supplement: Supplementary file 1 [file ijms-26-07882-s001.zip › ijms-3796309-supplementary.pdf]

Supplementary materials to MS

cAMP-Mediated Modulation of Functions of Green- and Blue-Sensitive Cones in Zebrafish

Darya A. Nikolaeva and Luba A. Astakhova

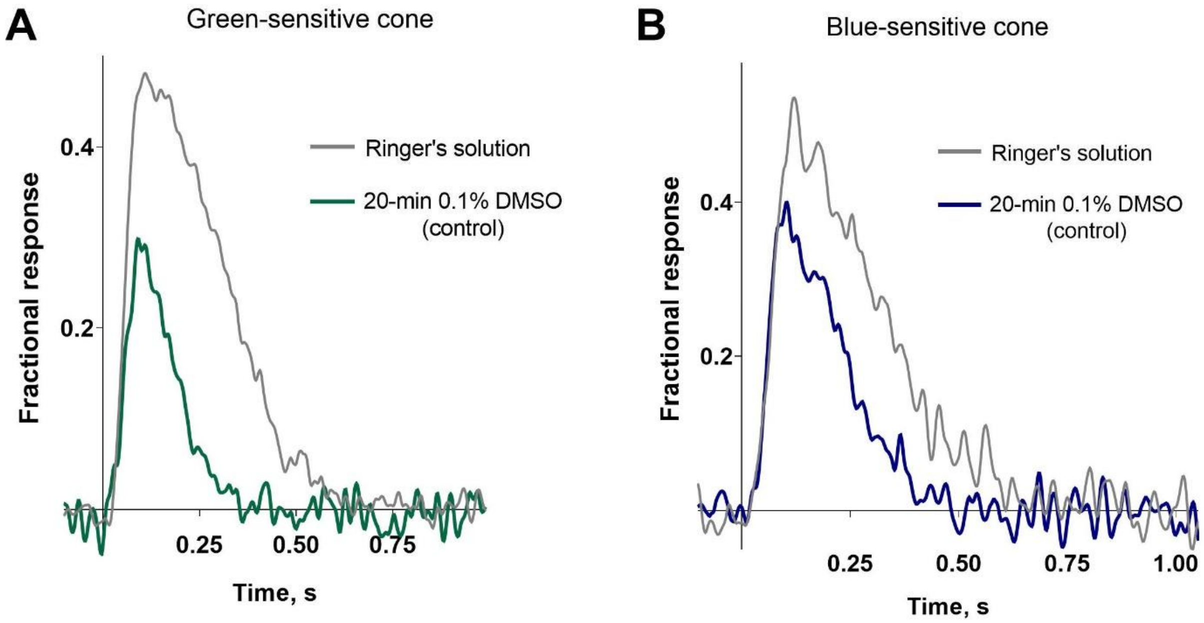

Figure S1. Typical examples of fractional non-saturated responses in normal Ringer's solution and after 20-min exposure of 0.1% DMSO (the control groups) for green- (A) and blue-sensitive cones (B). The intensity of the flashes for the responses in panel **A** were 56.3 photons / μm^2 per flash, for the responses in panel **B** were 56.3 photons / μm^2 per flash were 427 photons / μm^2 per flash, for both panels λ_{max} = 460 nm.

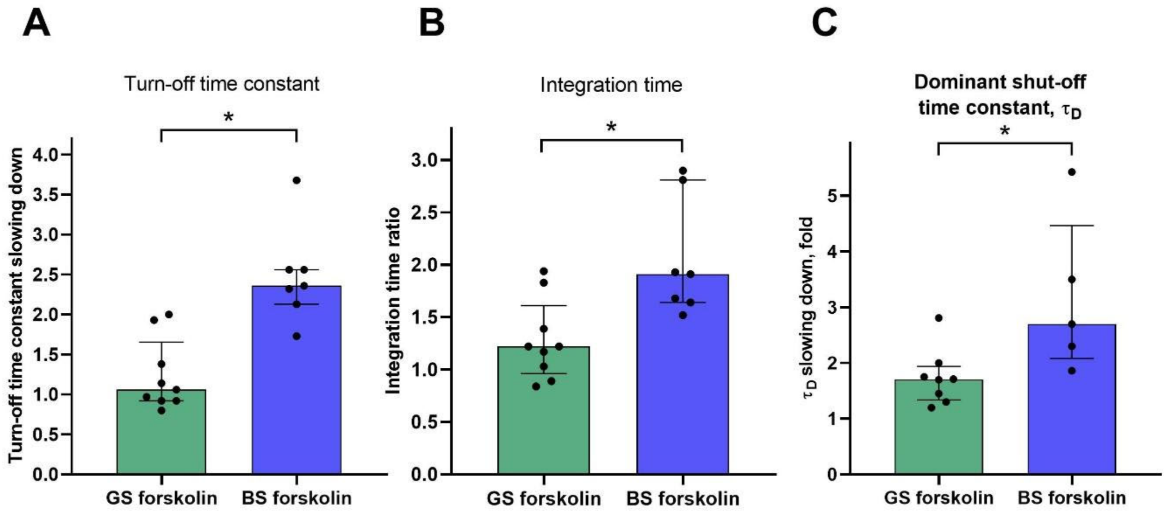

Figure S2. Direct comparison of the mean changes of turn-off rate (A) and integration time (B) of the non-saturated responses and dominant shut-off time constant for saturated responses (C) after forskolin exposure between green- and blue-sensitive cones. GS – green sensitive cones, BS – blue-sensitive cones. Data are shown as median ± interquartile range. The effects of forskolin on the turn-off rate and integration time of the unsaturated response and dominant shut-off time constant are statistically significantly more pronounced in blue-sensitive cones. Asterisk (*) indicates statistically significant difference between groups according to the Mann-Whitney test; $p = 0.0007$, Cohen's $d = 2.5$ for failling phase (A); $p = 0.012$, Cohen's $d = 1.6$ for integration time and $p = 0.019$, Cohen's $d = 1.7$ for τ_D slowing down)..
